# At the Cutting Edge or the Center of the Storm? Innovation in Public Health Through Health Promotion and Education in State Health Departments

**Published:** 2005-10-15

**Authors:** Randy Schwartz

**Affiliations:** American Cancer Society, New England Division

## Introduction

Health promotion and education components of state health agencies are at the center of the application of modern public health practice. Two reports on the future of public health, issued 15 years apart, detailed the changing nature and systemic problems of public health in the late 20th century (1) and identified the challenges in improving the public's health as we head into the 21st century (2). State health agencies — in collaboration with federal partners, community organizations, and health care systems — clearly play a critical role in ensuring the public's health. In response to these contemporary public health issues, the health promotion and education components of state health agencies have rapidly evolved, gaining the capacity to address newly recognized public health problems and become key players in ensuring that community and public health problems are addressed through cutting-edge public health strategies. These strategies include community mobilization, coalition building, and community-based interventions; integration of policy advocacy and media advocacy into comprehensive interventions; collaborations with academic institutions and other partners to advance the translation of research into practice; and the adoption of the social–ecological approach to public health interventions, in which the interplay of multiple interventions at multiple levels of society combine to provide the impact necessary to address deep problems.

This issue of *Preventing Chronic Disease* highlights several innovative health promotion and education initiatives conducted by state health agencies, showing not only the breadth of public health issues addressed by these agencies but also the scope of complex strategic issues undertaken by the leadership of these organizations.

## Historical Background

In the early 1980s, the health promotion and education units at state health agencies were small, often one-person programs, if present at all. With funding from the Health Education–Risk Reduction program, the growth of these enterprises began in earnest. Support from the Centers for Disease Control and Prevention's (CDC's) Center for Health Promotion and Education and the National Heart, Lung, and Blood Institute's (NHLBI's) National High Blood Pressure Education Program helped to build the capacity of the staff and provide program support for health promotion and education infrastructure. The Healthy People Objectives for the Nation (3) were embraced and became part of public health planning both nationally and in many states. Subsequent funding, including the Preventive Health and Health Services Block Grant, and various initiatives, such as cancer prevention and control, tobacco control, and injury prevention, contributed to both the capacity of the organization and the improvement of the public's health.

With the emergence of HIV/AIDS as a serious public health problem, public health education and school health became recognized as key components of an overall strategy that, when combined with disease prevention and control methods such as epidemiology, testing, and counseling, would strengthen an existing overall public health strategy. Development of public health programs in numerous areas such as environmental health and immunizations reinforced the integration of health promotion and education into comprehensive public health interventions. Professional organizations such as SOPHE (The Society for Public Health Education) and the Directors of Health Promotion and Education (DHPE), formerly the Association of State and Territorial Directors of Health Promotion and Public Health Education (ASTDHPPHE), contributed to the development of the professional staff and advocated for national- and state-level health promotion public policies and funding for health promotion programs.

## Challenges in Leadership Development and Strategic Priorities for Health Promotion

The health promotion and education leadership, staff, and programs initiated by state health agencies have synthesized and integrated the best of modern public health practice. In collaboration with the CDC, other federal agencies, and professional organizations, they have taken the best approaches available and worked with communities to implement them through state-level systems and public policies. Brief descriptions of the strategies used follow.

### Community mobilization and coalition building

Health promotion and education units have been pivotal in implementing key aspects of modern public health practice through community mobilization and with community participation, recognizing the capacity, politics, and culture of each community. Broad-based community health improvement initiatives, such as Colorado's privately run Healthy Communities, have recognized community-based coalition approaches as effective strategies for public health practice. Such coalition-based approaches are implemented by or in partnership with state health agency health promotion units. In many states, collaboration was enhanced by the work of the Turning Point initiative, funded by The Robert Wood Johnson Foundation and W.K. Kellogg Foundation (4), with its mission of strengthening the public health infrastructure and enhancing public health policy through community collaborations.

### Policy and media advocacy as essential elements of public health improvement

Fueled by work on tobacco prevention and control, health promotion and education intervention planning and delivery embraced the integration of policy advocacy and media advocacy as critical components of comprehensive community health improvement initiatives. The ASSIST program, funded by the National Cancer Institute, used standards that included both policy and media advocacy as essential to comprehensive tobacco-control program delivery (5). More recently, the CDC developed standards for comprehensive tobacco-control program delivery, recognizing the work of the ASSIST program and notable tobacco prevention and control programs in California and Massachusetts. Paradoxically, state health promotion programs that build the capacity of state and community stakeholders in policy and media advocacy should be prepared when these same trained advocates use their new skills to advocate to or even speak out against state health agencies.

### Collaboration with academic partners to accelerate the translation of research and evidence-based public health into practice

This issue of collaboration has received increasing attention over the past several years. The health promotion unit plays an invaluable role as a "linking agent" in the diffusion process (6). Links have been formed with CDC-funded Prevention Research Centers (PRCs) (7,8), Health Resources Services Administration-funded Public Health Training Centers, and other academic partners. State health promotion programs and staff must assume responsibility for enhancing practice and building capacity for the use of evidence-based practice by both program staff and constituents in community-based health organizations. Recently introduced tools such as *The Guide to Community Preventive Services* (9) and Cancer Control PLANET Web site (10) make this strategic imperative a more practical reality. Although some practitioners may, at times, seek to be told what to do or how to develop and implement an intervention through a "cookbook" approach, the skilled practitioner must integrate elements and skills of using a diagnostic approach, combined with best practices (11).

### Workforce development

Because of their varied backgrounds and experiences in the public health workforce (12), state health promotion leaders must step forward to develop the capacity and quality of not only health department staff skills but also the skills of community health workers practicing in community-based health organizations, local health departments, and other agencies of all sizes throughout the state. Again, in conjunction with academic partners and professional organizations such as local SOPHE chapters, health promotion workforce development remains both a need and a challenge.

### A remaining challenge: evaluation capacity building

Many of the interventions and applications of modern public health practices need to be measured for impact and outcome. State health promotion and education leaders can help address this challenge by incorporating evaluation into the agency at all levels but ensuring that the evaluation strategies are appropriate for the level of practice and staffing. Not all practitioners need to be evaluators, but all programs need monitoring and evaluation. The CDC Framework for Program Evaluation in Public Health (13) has been a valuable model to promote such integration. State health promotion leaders and the CDC, in conjunction with academic partners and professional organizations, must develop ways to more effectively build the evaluation capacity of staff and programs at all levels.

## Discussion

I have had the privilege of being a participant in the growth of health promotion and education in state health agencies as director of the Division of Community and Family Health at the state health agency in Maine for almost two decades prior to working at the American Cancer Society. The world of public health changed dramatically during that time, and health promotion practice rapidly evolved to meet such challenges. Partnership with and support by federal agencies, especially the CDC, and professional organizations, especially ASTDHPPE/DHPE and SOPHE, were essential to the division's continued quality and innovation with one goal in mind — improving the public's health. The collection of articles in this issue provides a glimpse into some of those innovative initiatives. These articles and others published in such journals as *Health Promotion Practice* help continually to advance the use of innovative and promising practices in real-world settings. The savvy practitioner can take the best of these examples and continue to evolve new and innovative approaches to their application in a variety of settings.
